# Clinical effectiveness, quality of life and cost-effectiveness of Flaminal® versus Flamazine® in the treatment of partial thickness burns: study protocol for a randomized controlled trial

**DOI:** 10.1186/s13063-016-1240-5

**Published:** 2016-03-05

**Authors:** Zjir M. Rashaan, Pieta Krijnen, M. Elske van den Akker- van Marle, Margriet E. van Baar, Adrianus F. P. Vloemans, Jan Dokter, Fenike R. H. Tempelman, Cees H. van der Vlies, Roelf S. Breederveld

**Affiliations:** Department of Surgery, Leiden University Medical Center, Postbus 9600, 2333 ZA Leiden, The Netherlands; Burn Centre, Red Cross Hospital Beverwijk, Postbus 1074, 1942 LE Beverwijk, The Netherlands; Department of Medical Decision Making, Leiden University Medical Centre, PO 9600, 2333 ZA Leiden, The Netherlands; Association of Dutch Burn Centres, Postbus 1015, 1940 EA Beverwijk, The Netherlands; Burn Centre, Maasstad Hospital, PO Box 9100, 3079 DZ Rotterdam, The Netherlands

**Keywords:** Flaminal®, Silver sulfadiazine, SSD, Flamazine®, Partial thickness burns, Burns

## Abstract

**Background:**

Partial thickness burns are painful, difficult to manage and can have a negative effect on quality of life through scarring, permanent disfigurement and loss of function. The aim of burn treatment in partial thickness burns is to save lives, stimulate wound healing by creating an optimumly moist wound environment, to have debriding and analgesic effects, protect the wound from infection and be convenient for the patient and caregivers. However, there is no consensus on the optimal treatment of partial thickness wounds. Flaminal**®** and Flamazine**®** are two standard treatment options that provide the above mentioned properties in burn treatment. Nevertheless, no randomized controlled study has yet compared these two common treatment modalities in partial thickness burns. Thus, the aim of this study is to evaluate the clinical effectiveness, quality of life and cost-effectiveness of Flaminal**®** versus Flamazine**®** in the treatment of partial thickness burns.

**Methods/Design:**

In this two-arm open multi-center randomized controlled trial, 90 patients will be randomized between Flaminal**®** and Flamazine**®** and followed for 12 months. The study population will consist of competent or temporarily non-competent (because of sedation and/or intubation) patients, 18 years of age or older, with acute partial thickness burns and a total body surface area (TBSA) of less than 30 %. The main study outcome is time to complete re-epithelialization (greater than 95 %). Secondary outcome measures include need for grafting, wound colonization/infection, number of dressing changes, pain and anxiety, scar formation, health-related quality of life (HRQoL), and costs.

**Discussion:**

This study will contribute to the optimal treatment of patients with partial thickness burn wounds and will provide evidence on the (cost-)effectiveness and quality of life of Flaminal**®** versus Flamazine**®** in the treatment of partial thickness burns.

**Trial registration:**

Netherlands Trial Register NTR4486, registered on 2 April 2014.

## Background

Partial thickness burn wounds are painful, difficult to manage, and susceptible to infection [1]. The aim of burn treatment in partial thickness burns is to promote rapid wound healing, decrease pain and suffering, protect the wound from infection, minimize scar formation and functional impairment, and to enable patients to return to normal daily activities as soon as possible [2, 3]. Thus, an ideal dressing should stimulate wound healing by creating an optimumly moist wound environment, but also have a debriding and analgesic effect. Moreover, an ideal dressing should protect the wound from infection and be convenient for the patient and caregivers.

Nowadays, many topical dressing materials are available for the treatment of superficial and deep partial thickness burns, while there is no strong evidence to support their use [4]. In clinical practice, silver-containing dressings and topicals, in particular silver sulfadiazine (SSD), have been the most commonly used burn wound dressing in the treatment of partial thickness burns for over 30 years [4–8].

The popularity of SSD can mainly be explained by its antimicrobial effect in vitro against a wide range of gram-positive and gram-negative microorganisms including resistant forms such as methicillin-resistant *Staphylococcus aureus* (MRSA) and vancomycin-resistant *Enterococcus* (VRE), and against fungi and anaerobes [9–11]. However, a Cochrane review of 26 randomized controlled trials (RCTs) found insufficient evidence to establish whether silver-containing dressings or topical agents prevent wound infection more effectively compared to non-silver containing treatments [12].

Several studies have shown that silver is highly toxic to both keratinocytes and fibroblasts in in vitro models [7, 13–15]. Other studies found that SSD will form a pseudoeschar which can promote bacterial proliferation and requires frequent removal or debridement on a daily basis to facilitate re-epithelialization and the optimal assessment of the wound state. In contrast, two other studies found that some silver preparations are not toxic and suggest positive effects of silver on wound healing [16, 17]. A number of systematic reviews of clinical trials showed that SDD is consistently associated with poorer wound healing than non-silver treatment for superficial and partial thickness burns [4, 18, 19]. However, the results of these reviews should be interpreted with caution because of the high risk of bias in these clinical trials. With regard to costs, a number of studies suggested that SDD is less cost-effective than Mepilex® Ag, Aquacel® Ag and Acticoat® [20–24].

Recently, Flaminal® (Flen Pharma, Kontich, Belgium) was introduced onto the market. Flaminal® consists of hydrated alginates polymers in a polyethyleneglycol (PEG) matrix embedded with a biologic enzyme system based on glucose oxidase and lactoperoxidase that are stabilized by guaiacol [15]. This enzyme system forms free radicals which destroy the cell wall of absorbed bacteria. Furthermore, short-chain PEG dissolves dry scab and necrotic material which results in lysed material. Wound exudate, including bacteria, and lysed material is absorbed by alginates in hydrated form. These two steps result in continuous debridement [25, 26].

Pre-clinical data by Vandenbulcke et al. and de Smet et al. showed that Flaminal® Forte is not toxic to keratinocytes and fibroblasts in vitro [15, 27]. In effect, Flaminal® Forte may not damage skin cells which will finally result in undelayed wound healing. Two studies have indicated a faster wound healing when a partial thickness burn wound was treated with Flaminal® Forte compared to SSD [28, 29]. But these results should be interpreted with caution due to the retrospective design of these studies. However, a shorter wound healing time would not only reduce length of hospital stay (LOS) but probably also scar formation since Deitch et al. and Cubison et al. demonstrated that burn wounds which heal within 21 days have less risk of developing hypertrophic scars and contractures [1, 30].

There are studies showing conflicting data with respect to bacterial growth under Flaminal® Forte; with some demonstrating count reductions [15, 27] while others show increases [29].

Dressings with SSD should be changed every 24 hours, and removing the pseudoeschar, which is a result of the SSD base drying out, is usually painful. In contrast, Flaminal® Forte can be applied every other day after the third post-burn day (PBD) and can be removed easily from the wound. However, the effect on pain perception and anxiety during dressing changes between these treatments have not yet been compared. Also, the effect of both treatments on LOS, scarring and health-related quality of life (HRQoL) are not known.

Finally, medical costs including those for medical staff, materials for wound care, surgical procedures, hospital stay, HRQoL, and productivity loss due to the burn injury are unknown for both treatments. A cost-effectiveness analysis is thus mandatory to evaluate the long-term health economic outcomes of the studied treatments and justify the application of both treatments in clinical practice.

### Objectives

The aim of this study is to evaluate the clinical effectiveness, quality of life and cost-effectiveness of Flaminal**®** versus Flamazine**®** (SSD) in the treatment of partial thickness burns.

## Method/Design

This investigator initiated, open label, multi-center, RCT compares the effects of treatment and cost-effectiveness of Flaminal**®** Forte versus Flamazine**®** in the treatment of partial thickens burns.

### Study population

The study will be performed in two of the three burn centers (Beverwijk and Rotterdam) in The Netherlands. In these burn centers, both Flaminal® and Flamazine® are therapeutic options for treating partial thickness burns. Patients who are admitted to the Beverwijk or Rotterdam Burn Centre and who meet the following inclusion criteria will be eligible for this study: competent or temporarily non-competent (because of sedation and/or intubation); partial thickness burns of minimally 1 % total body surface area (TBSA) (possibly in combination with full thickness burns); hospital admission within 48 hours of burn injury; written informed consent by the patient. Patients meeting one or more of the following criteria are excluded: age below 18 years; TBSA of more than 30 %; burns caused by chemicals, electricity or radiation; if local therapy with a topical agent has already started; patients who are expected (according to the treating medical physician) to be non-compliant with the study protocol.

### Recruitment, consent and randomization

All patients who are admitted to the burn center undergo standardized screening and baseline procedures according to the local protocol. The local investigator will check the inclusion and exclusion criteria of the study and will inform the participant about the study. If the participants are willing to participate in this study, they must provide written informed consent. If an eligible patient is temporarily non-competent because of sedation and/or intubation, a legal representative of the patient, according to the Dutch Medical Treatment Act (WGBO), will be informed about the study and will provide written informed consent. After the sedation or intubation has ended, the patient will be asked to confirm willingness to participate in the study in writing, otherwise the participation is discontinued and the collected study data will be destroyed.

After informed consent has been obtained, the largest partial thickness area will be assigned as the study area. Thereafter, the patient will be randomly assigned to one of the two study arms using TenALEA (Trans European Network for Clinical Trials Services), an online randomization program (www.flam-studie.nl). The online-randomization is stratified by center and uses variably sized blocks in a 1:1 ratio. In both participating hospitals the local trial coordinator will receive a username and password for online randomization. After randomization the local trial coordinator of that center and the central trial coordinator will receive an email with the inclusion number and the randomization outcome. The outcome will also be displayed on the website, only visible for the randomizing local and central trial coordinators. Then, all the burn wounds will be treated with the treatment that is assigned by the randomization.

When the study area that was initially assessed as a partial thickness burn, turns out to be a full thickness burn area after performing laser Doppler imaging (LDI), then the study area will be replaced: the second largest partial thickness area (confirmed by LDI) will then be chosen as the study area.

For practical reasons it is not possible to blind the patients. It is also impossible to blind the medical staff who provide the burn wound care, because they are involved in all aspects of the care and are able to recognize each treatment from its appearance. No blinding of other outcomes is also possible for the same reasons.

### Interventions

The patient will be allocated to one of the following treatments:

- o*Flaminal® Forte:* treatment with Flaminal® Forte (glucose oxidase-lactoperoxidase guaiacol complex of 50 g in 5.5 % alginogel) will be initiated within 24 hours after admission. Before applying Flaminal® Forte on the wound, pain medication must be given. Paracetamol, Oxynorm and Oxycontin will be used as standard pain medication. The usage and doses of pain medication will be monitored. The burn wound will be cleaned and rinsed with Prontosan® followed by careful dabbing and drying of the wound. The wound will then be covered with a non-adhesive dressing on which a sufficiently thick layer (4–5 mm) of Flaminal® Forte has been applied. A net bandage/dressing will be used to keep the dressing in place. Dressing changes will be performed daily during the first 3 days post burn and thereafter every other day. If an infection is suspected, or in case of leaking or insufficient gel, the dressing with Flaminal® Forte may be changed daily after 3 days post burn. In case of wound colonization or infection, treatment with Flaminal® Forte will be changed to another relevant treatment based on the results of the wound culture
- o*Flamazine®:* treatment with Flamazine® (silver sulfadiazine 10 mg/g in hydrophilic crème base) will consist of daily washing and application of Flamazine®. Before applying Flamazine® on the wound, pain medication must be given. Paracetamol, Oxynorm and Oxycontin will be used as standard pain medication. The usage and doses of pain medication will be monitored. The burn wound will be cleaned and rinsed with Prontosan® followed by carefully dabbing and drying of the wound. A sufficiently thick layer (at least 2–3 mm) of Flamazine® will be applied directly on the wound. The cream will be covered with a non-adhesive dressing. A net bandage/dressing will be used to keep the dressing in place. This procedure is repeated once every 24 hours until the sixth day post burn. Thereafter, the treatment of all patients in this study arm will consist of Furacine Soluble Dressing (Furacine 2 mg/g ointment) on the seventh day post urn, and Flamazine® on the eighth day post burn. Treatment with Furacine Soluble Dressing and Flamazine® will be alternated until complete wound healing/operation because of the cytotoxicity of the silver particles in Flamazine® on the wound bed when used continuously. In case of wound colonization or infection the treatment will be replaced with another relevant treatment based on the results of the wound culture

After discharge, patients in both group will be treated in an outpatient setting, according to the local protocol.

### Outcome measures

#### Primary outcome

Primary endpoint is time to complete re-epithelialization (greater than 95 %) of the study area, in days, judged by two experienced burn specialists during each dressing change. Complete re-epithelialization of the study area is only affirmed when the two burn specialists agree with each other.

#### Secondary outcomes/study parameters

- o*Clinical outcomes:*
  - Need for operation which is evaluated between 10 and 14 days post burn. Reasons for operation are that the experienced burn specialist expects no further wound healing in the next 7 to 11 days of the partial thickness area or a full thickness burn. If the decision to graft is made before 10 to 14 days post burn then the operation will still performed before 10^th^ day post burn day. The only indication for operation before the 10^th^ PBD is when a partial thickness burn becomes a full partial thickness burn. The treatment of full thickness burns is split skin graft in an early stage
  - Percentage TBSA of the study area that covered with skin graft
  - Colonization: twice a week a wound swab will be taken from the study area. The wound swab will then be sent for laboratory investigation. In brief, the analysis of the wound swab will include the following steps. The microscopic examination will entail gram-staining. Thereafter, a quantity of the specimen will be cultured to obtain a pure single specimen culture. Finally, the sensitivity of the organisms to specific local therapy will be determined. In case of wound colonization the treatment will be changed to another relevant treatment based on the results of the wound culture
  - Infection: infection is suspected if a combination of skin redness, pain, swelling, tenderness, warmth, fever or pus draining from the wound is present. Infection is a clinical evaluation of the wound, in presence of absence of positive wound culture, and is judged by a physician at the burn center during each dressing change
  - Number of dressing changes
  - Use of systemic antibiotics

- o*Patient-reported outcomes:*
  - Pain will be measured during the application and removal of the wound dressing (procedural pain, measured directly after dressing change) and background pain (measured in the morning and evening). Pain will be measured twice daily during hospital admission by use of a Visual Analogue Thermometer (VAT), which is a numeric scale from 0 (no pain) to 10 (worst imaginable pain)
  - Itching will be measured daily in the evening during hospital admission by use of a VAT [31]
  - Anxiety: the Burn Specific Pain Anxiety Scale (BSPAS) will be scored on day 7 ± 2 post burn and on the day of discharge. The BSPAS is a valid and reliable nine-item self-report scale for the assessment of pain-related and anticipatory anxiety in burned patients [32, 33]
  - Health-related quality of life (HRQoL) will be measured using the following questionnaires, in the week before discharge and at 3, 6 and 12 months post burn:

1. Burn-specific quality of life (QoL) will be measured using the Dutch version of the Burn Specific Health Scale (BSHS)–Brief [34, 35]. The BSHS-Brief is a valid and reliable self-administered questionnaire that covers nine domains (heat sensitivity, affect, hand function, treatment regimens, work, sexuality, interpersonal relationships, simple abilities, and body image). The questionnaire takes 10–15 minutes to complete and 5 minutes to score. Responses are scored by the patient on a five-point scale from 0 (extreme) to 4 (none/not at all) for each of the 40 items. Mean scores are calculated for each of the domains
2. General HRQoL will be measured using the EuroQol-5D (EQ-5D) questionnaire [36]. This simple and generic description of health status is widely used in studies on clinical and economic appraisal. The EQ-5D outcomes will be converted in utility scores between 0 (death) and 1 (perfect health) based on empirical valuations [37]. From the area under the utility curve during the 12 months of follow-up quality- adjusted life years (QALYs) will be calculated
  o*Scar formation*: the following aspects of scar formation will be measured after 3, 6 and 12 months post burn:
    Scarring will be measured using the Patient and Observer Scar Assessment Score (POSAS), a valid and reliable scale that is designed for the evaluation of all types of scars by professionals and patients. It consists of two numeric scales: the Patient Scar Assessment Scale which is completed by the patient and the Observer Scar Assessment Scale which is completed by the medical staff. The variables scored by the patient are pain, itching, color, stiffness, thickness and irregularity [38–40]. The variables scored by the medical staff include vascularization, pigmentation, thickness, relief and pliability
    Elasticity will be measured using a Cutometer (Courage & Khazak, Köln, Germany), a validated instrument to measure the vertical deformation of the skin in millimetres when the skin is pulled by means of a controlled vacuum into a circular aperture [41]
    Scar color and pigmentation will be measured using the DSM II colorimeter (Dermaspectrometer).This is a validated instrument to measure scar color by a narrow-band simple reflectance meter [41]
  o*Total (medical and non-medical) costs:* total costs in this study represent direct health care costs (inpatient and outpatient medical costs), direct non-healthcare costs and indirect non-health care costs (productivity loss). Personnel time and used materials for wound care and surgical procedures, hospital days for initial stay and re-admittance and outpatient visits during the first year will be measured prospectively as part of the case record form. Health resource use outside the hospital, travel costs and productivity losses will be recorded by questionnaires filled out by the patients at 3, 6 and 12 months post burn. Cost of dressing changes and surgical procedures will be assessed by translating the personnel time and used materials into costs by means of gross salaries and market prices. The costs of hospital stay and outpatient care in burn centers will be calculated by multiplying the number of hospital days respectively outpatient visits with their cost prices [42]. Other healthcare use will be translated into costs by standard prices [43]. Productivity losses will be valued using the friction cost method [44]
  o*Baseline parameters:* age, gender, skin type, wound etiology, bacterial contamination at admission, location of the wound, type of wound, TBSA% and co-morbidities. In all patients, the burn depth of the study area will be accurately determined on day 2–5 post burn by clinical evaluation and LDI scan using the moorLDI2-Burn Imager™ (Moor Instruments, Axminster, UK) and, based on pre-defined criteria (Table 1), be classified as superficial, intermediate or deep partial thickness injury. The size of each burn wound is then estimated in TBSA. In a LDI the low-intensity laser beam is scanned across a tissue surface in a raster fashion using a moving mirror. There is no direct contact with the tissue being assessed. The wounds are scanned by a trained research physician or nurse, after removal of topical medication (during regular wound treatment). All research physicians and nurses in both burn centers have followed the same training sessions and have the same experience. The scanning will take 1–5 minutes.

**Table 1:** Clinical assessment and laser Doppler imaging (LDI) results

Classification	Clinical properties						LDI color
	Blisters	Color/Appearance	Pliability	Capillary refill	Pain	Healing time	
Superficial partial thickness burns	Small blister: intact and open	Pink-red, shiny	Supple	<2 sec	++	Within 14 days	Red
Intermediate partial thickness burns	Blisters: intact and open	Pink-red, shiny and dry	Mix of supple and stiff	<2 sec	+	14–21 days	Yellow
Deep partial thickness burns	Blisters: intact and open	Red, shiny and dry	Mix of supple and stiff	>2 sec	+/-	>21 days or even no spontaneous healing	Blue
Full thickness burns	None	White-yellow, red, brown and black	Stiff	>2 sec	-	No spontaneous healing	Blue

A schedule with baseline and outcome measurements during the study is presented in Table 2.

**Table 2:** Time schedule for study procedures and assessments

Procedure/Assessment	Admission	Treatment phase	Follow-up (month)		
			3	6	12
Standard screening, baseline procedures	x				
Check eligibility (inclusion criteria)	x				
Provide patient Information	x				
Obtain written informed consent	x	x (confirmation by patients who were initially non-competent)			
Randomization (Flaminal® vs Flamazine®)	x				
Baseline parameters	x				
LDI		x			
Wound colonization	x	x			
Wound healing		At each dressing change			
Need for surgery of partial and/or full thickness burns and % of study area requiring skin graft		10–14 PBD			
Colonization	x	Swabs on Mondays and Thursdays			
Infection	x	Clinical judgment during dressing changes			
Dressing changes	x				
Use of systemic antibiotics	x				
Pain and anxiety					
Visual analogue thermometer (VAT)	x	Daily, before and after dressing change and in the evening			
Itching	x	Daily in the evening on 7^th^ ±2 PBD and day of discharge			
Burn Specific Pain and Anxiety Score (BSPAS)	x				
Health-related quality of life (HRQoL)		In the last week of hospitalization			
Burn Specific Health Scale Brief (BSHS-Brief)		In the last week of hospitalization	x	x	x
EuroQol-5D (EQ-5D)			x	x	x
Scarring					
POSAS			x	x	x
Cutometry			x	x	x
Dermaspectrometry			x	x	x
Costs		Daily	x	x	x

*LDI* laser Doppler imaging, *PBD* post-burn day

### Sample size calculation

The study is designed to demonstrate a clinically relevant difference regarding time to complete epithelialization between the treatment arms. Based on a retrospective study of 70 patients with superficial and deep partial thickness burns of the hand, we expect wounds to heal in 11 days on average with Flamazine**®** and in 6 days on average with Flaminal**®** (pooled standard deviation 7.5 days) [28]. With alpha set at 5 %, 41 patients are needed in both intervention arms to detect a difference in wound healing time with 80 % power. To allow for 10 % attrition, a total of 90 patients will be included in the study.

### Statistical analysis

The analysis will be performed according to the intention-to-treat principle. The baseline patient characteristics will be described as mean ± standard deviation for normally distributed continuous variables, as median (range) for skewed continuous variables, and as number (proportion) for categorical variables. Differences in time to complete re-epithelialization will be compared in both treatment groups will be analyzed with Kaplan-Meier curves and log rank test.

If the baseline characteristics seem unbalanced despite randomization, a multivariable Cox regression analysis will be performed to correct for potentially confounding variables to confirm the primary analysis.

The secondary clinical and patient-reported outcomes on specific follow-up moments will be compared between the treatment groups using the two-sided *t* test or Mann-Whitney test for continuous data, and a two-sided Chi-square test or Fisher’s exact test for categorical data. Repeatedly measured study parameters such as pain and quality of life will be also analyzed using a linear mixed model with treatment as fixed effect and patient as random effect. In the analyses, *p* values <0.05 will be considered statistically significant.

### Economic evaluation

The economic analysis will be performed from the societal perspective. The follow-up period is 12 months. Due to this short time frame, no discounting will take place. Cost-effectiveness ratios will be calculated by dividing the difference in average costs per patient between both intervention groups by the difference in primary outcome (time to complete re-epithelialization). Dressing changes are reported to be one of the most painful and traumatic aspects of burn treatment. This impact is not measured in the primary outcome measure (time to greater than 95 % re-epithelialization). In a sensitivity analysis, therefore, the difference in costs will be related to the difference in dressing changes between both treatments.

### Ethical consideration and safety

This study has been approved by the Medical Research Ethics Committee of Noord-Holland (NL43671.094.13). EudraCt number: 2013-000901-21. This study is also registered in the Netherlands Trial Registry (NTR), trial number 4486 and will be conducted in agreement with the Declaration of Helsinki, version Fortaleza (Brazil), October 2013, concerning the Ethical Principles for Medical Research Involving Human Subjects, and in accordance with the International Conference on Harmonization (ICH) of Good Clinical Practice (GCP) Guidelines and the valid Dutch laws. Adverse events (AEs), serious adverse events (SAEs) and suspected unexpected serious adverse reactions (SUSARs) are documented and reported to the competent authorities. All AEs will be followed until they have abated, or until a stable situation has been reached. Depending on the event, follow-up may require additional tests or medical procedures as indicated, and/or referral to the general physician or a medical specialist. There is no additional risk or discomfort for the patients in this study compared to daily practice. Since most of the measurements and questionnaires used in the study are also implemented in daily care of burn patients in the Dutch burn centers, participation does not involve a large extra burden for them.

## Discussion

This randomized controlled study will enable a comparison of the effectiveness, cost-effectiveness and quality of life of Flaminal**®** and Flamazine**®**, two common treatment modalities for partial thickness burns.

An accurate diagnosis of the partial thickness burns is essential in our study. Several studies have shown that clinical evaluation of burn depth is highly dependent on the experience of the clinician and that experienced clinicians are accurate in about 50–75 % of the cases [45–49]. Therefore, we use LDI, which has an accuracy of 95 % in combination with clinical evaluation of the wound, for measuring burn depth [49–51]. Furthermore, in our study two experienced wound specialists must agree on the time to complete re-epithelialization in order to optimize the accuracy of wound re-epithelialization. Bloemen et al. have shown that experienced observers are able to evaluate the re-epithelialization rate in a reliable and effective way [52]. No digital analysis is required to evaluate wound re-epithelialization since clinical evaluation by an experienced burn specialist is as equally effective as digital analysis [53].

This study will contribute to optimize the treatment of patients with partial thickness burn wounds from both the clinical and economical perspective.

### Trial status

Recruitment started in February 2014, and was completed on 20 October 2015. The follow-up of included patients is ongoing. The planned end date of the data collection is November 2016.

## Abbreviations

GCP: Good Clinical Practice
GLG: glucose oxidase combined with lactoperoxidase, stabilized by guaiacol
METC: Medical Research Ethics Committee (MREC); in Dutch: medisch ethische toetsing commissie (METC)
PBD: post-burn day
PEG: hydrated alginates polymers in a polyethyleneglycol
POSAS: Patient and Observer Scar Assessment Score
RCT: randomized controlled trial
SSD: silver sulphadiazine
TBSA: total body surface area
